# Variation in Odour Profiles of Cauliflower, Curly Kale and Broccoli (*Brassica oleracea* L.) Cultivars Is Affected More by Genotype Rather than Herbivore Feeding

**DOI:** 10.3390/plants14071014

**Published:** 2025-03-24

**Authors:** Raimondas Mozūraitis, Peter Hambäck, Anna-Karin Borg-Karlson, Richard James Hopkins

**Affiliations:** 1 Laboratory of Chemical and Behavioural Ecology, Institute of Ecology, Nature Research Center, Akademijos 2, LT-08412 Vilnius, Lithuania; 2 Department of Zoology, Stockholm University, SE-10691 Stockholm, Sweden; 3 Department of Ecology, Environment and Plant Sciences, Stockholm University, SE-10691 Stockholm, Sweden; peter.hamback@su.se; 4 Division of Organic Chemistry, Department of Chemistry, School of Engineering Sciences in Chemistry, Biotechnology and Health, Royal Institute of Technology, SE-10044 Stockholm, Sweden; akbkeko@gmail.com; 5 Independent Consultant in Research and Development, Sunnersbol 21, SE-74794 Alunda, Sweden; richardjameshopkins1967@gmail.com

**Keywords:** *Brassica oleracea*, headspace, volatile organic compounds, induced volatiles

## Abstract

Volatile plant compounds are essential for host plant selection by herbivores and particularly important for the behaviour of parasitoids seeking larvae in which to lay eggs. Headspace extracts were collected from intact plants of four *Brassica oleracea* genotypes, as well as from plants damaged by larvae of *Mamestra brassicae* or *Pieris rapae*. In total, 52 volatiles present in the headspaces of four genotypes were selected for multivariate analyses. The most abundant groups of volatiles were terpenes and esters, represented by 20 and 14 compounds, respectively. The qualitative and quantitative differences in odour profiles between the four genotypes were sufficient to differentiate between groups using multivariate analysis techniques. The most distinct volatile blends originated from curly kale, followed by cabbage, cauliflower and broccoli. Multivariate analysis revealed that genotypes affected the composition of the volatile blends to a large extent compared to the herbivore damage by the different species tested. In curly kale, broccoli and cauliflower, the differences in odour bouquets were more expressed between plants with and without active feeding, independent of the herbivore identity, while in cabbage, larger differences were observed between odour profiles with different herbivore feedings, independent of whether the herbivore was present or removed.

## 1. Introduction

Plants from the Brassicaceae family play a significant role in worldwide vegetable production in terms of tons produced/consumed, ranking second after Solanaceae [1,2]. However, insect pests pose a great challenge to *Brassica* crop production worldwide. Over 20 insect pest species are *Brassica* specialists and cause crop losses at economically significant levels [3,4]. Whilst the demand for organically produced vegetables gradually increases, profitability is relatively low due to high losses from insect pests. Lepidoptera (butterfly and moth) larvae, in particular, leave large holes in the plant, contaminate the crop with frass and are highly visible themselves. The development of cropping systems that result in a reduction in plant damage while at the same time being easy to manage could contribute substantially to a decrease in losses in organic vegetable production. Cropping system diversification by selecting optimal spatial arrangements of attractive and less attractive genotypes influences attack rates of pest insects on the preferred type of plant [5,6,7]. It was shown that the number of eggs on a preferred plant is decreased only when the plant is growing in close proximity to a less preferred plant [6,8]. Predictions of the influence of vegetation complexity on herbivore abundance are difficult because of scarce knowledge of interacting mechanisms in diverse habitats in the field and herbivore reaction specificity [9,10].

One of the mechanisms explaining reduced pest pressure in diversified plantings is the decreased ability of pests to locate the preferred type of cultivar, as proposed by the resource concentration hypothesis [11]. Visual characteristics, including shape, size and colour, as well as olfactory cues comprised of attractants and repellents, are essential when searching for host plants [12]. Crop genotypes emit variable blends of volatile organic compounds (VOCs), which have behaviour-modifying effects on pests, predators and parasitoids. The odours emitted by herbivore-damaged plants are particularly important to parasitoids seeking herbivore larvae to lay eggs [13,14]. By knowing the composition and behavioural effects of volatiles in these blends, we could predict which genotypes are more or less likely to be targeted by pests or attract their natural enemies. This is crucial for effective pest management, especially in diversified plantings.

The small white butterfly, *Pieris rapae* L. (Lepidoptera: Pieridae) and the cabbage moth, *Mamestra brassicae* (L.) (Lepidoptera: Noctuidae), are serious pests of cruciferous crops, including *Brassica oleracea* L. (Brassicales: Brassicaceae) [15]. *P. rapae* is a crucifer-feeding specialist [16]. Larvae primarily damage leaves, causing substantial damage by skeletonising foliage [17]. *M. brassicae* has a broader host range and is a polyphagous species [18]. Contrary to *P. rapae* species, at the last instars, larvae of *M. brassicae* bore into the heads of cabbage or cauliflower, leading to extensive damage [19]. Both species have a wide geographical distribution, have adapted to various climates and can cause significant economic losses in agriculture due to their feeding habits and rapid reproduction rates [20].

Some studies have shown that *P. rapae* is able to differentiate *B. oleracea* genotypes from a small distance and that the host selection behaviour is strongly dependent on spatial heterogeneity of cultivars in field experiments [21]. Adult female *P. rapae* can also distinguish colours and preferably land on green objects [22]. However, Ikeura et al. have demonstrated that visual cues alone were insufficient for *P. rapae* to differentiate between cabbage and non-host plant lettuce [23]. Only when the transparent polyethylene bags had holes (visual cues complimented by olfactory signals) did female adults have a clear preference for landing on cabbage.

Taking into consideration the importance of volatile phytochemical cues for host location by herbivores and the detection of herbivores by their natural enemies, this research examines the variation in the plant odour profiles of *B. oleracea* in the context of genotypic variation and the variation induced by herbivore damage. We did this by examining the odour profiles of undamaged cabbage *B. oleracea* subs. *capitata*, cv. Consul, cauliflower *B. oleracea* subs. *botrytis*, cv. Nautilus, curly kale *B. oleracea* subs. *acephala*, cv. Winterbor and broccoli *B. oleracea* subs. *italica*, cv. Marathon plants and from plants damaged by two important Lepidoptera pest species, the small white butterfly, *P. rapae* and the cabbage moth*, M. brassicae*. The *B. oleracea* genotypes were selected based on our previous work [21] and their widespread use among commercial growers.

## 2. Results

### 2.1. Chemical Composition of Plant Volatiles

Gas chromatographic–mass spectrometric (GC–MS) analyses of plant volatiles revealed 52 compounds that were only present or occurred in significantly large amounts in the headspace samples obtained from four intact *B. oleraceae* cultivars, as well as plants at different stages of infestation by two Lepidoptera herbivore species, compared to those of blank samples. These compounds were attributed to eight groups. The majority of VOCs were terpenoids biosynthesised by the isoprenoid pathway, including nine monoterpenes, five oxygenated monoterpenes, eleven sesquiterpenes and two homoterpenes. Another large group was comprised of fourteen esters. The remaining compounds were three sulphides, one isothiocyanate and seven volatiles with different functional groups that were allocated to the group named other chemicals in Table 1.

### 2.2. Variation in the Odour Profiles of Intact Plants

**Figure 1:**
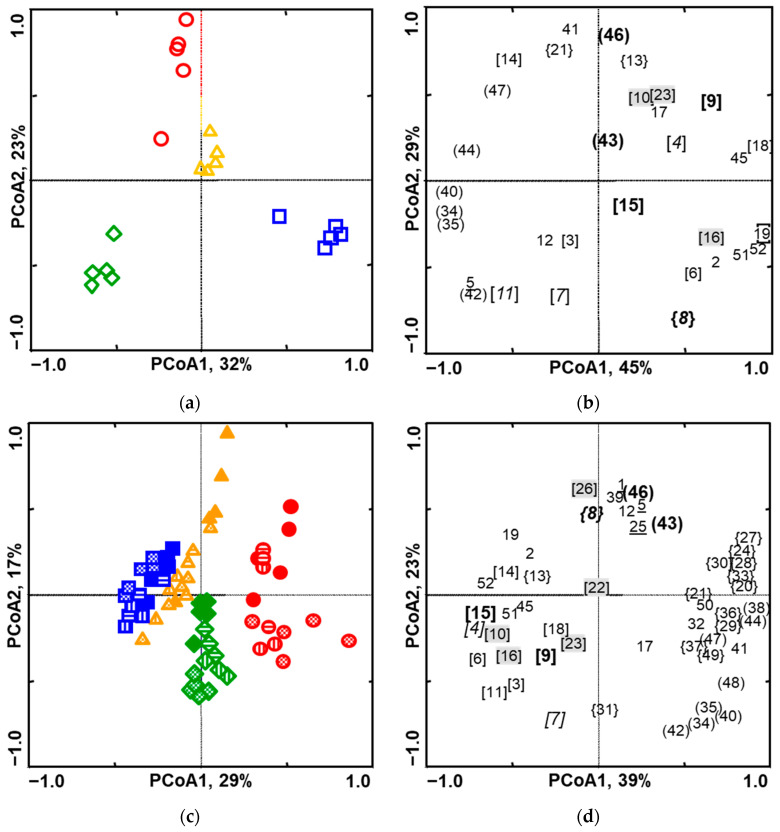

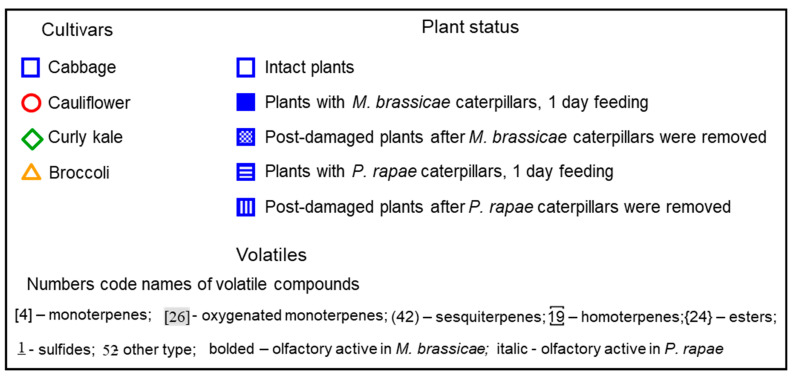
Plots derived from principal coordinate analysis of 52 volatiles released by four *Brassica oleraceae* cultivars of intact (**a**,**b**) and infested with caterpillars of either *Mamestra brassicae* or *Pieris rapae* species and post-damaged stages (**c**,**d**). Distribution of cases, i.e., odour bouquets of brassica cultivars, are presented in plots (**a**,**c**); distribution of variables, i.e., volatile compounds are shown in plots (**b**,**d**); PCoA stands for principal coordinate axis; values (eigenvalues) presented after PCoA name indicates the percent of variation explained by certain PCoA in (**a**,**c**) while corresponding values in plots (**b**,**d**) show the percent of associations between cases and variables explained by certain PCoA.

In total, 20, 24, 25 and 19 individual volatile compounds were collected from intact cabbage, cauliflower, curly kale and broccoli plants, respectively. Principal coordinate analyses (PCoAs) conducted on the obtained data revealed that all four varieties were separated from each other. The first PCoA axis accounted for 32% of the total variation. It correlated with relative amounts of volatiles, which contributed to the separation between specimens of cabbage and curly kale as well as groups of cauliflower and broccoli. The second PCoA axis contained 23% of the total variation and explained the separation between cauliflower and broccoli as well as groups of curly kale and cabbage (Figure 1a).

The specimens of cabbage formed a cluster projected into the quarter formed by the positive side of the 1st and the negative side of the 2nd PCoA axis (Figure 1a) due to high values of compounds that formed a group consisting of sabinene (6), (*E*)-sabinene hydrate (16), DMNT (19) unknowns (2, 51, 52) and neighbouring (*Z*)-3-hexenyl acetate (8) (Figure 1b). The values of these compounds in the blends collected from the cabbage plants differ significantly from those of other cultivars. Unidentified (45), *p*-mentha-2,4(8)-diene (18) and α-terpinene (9) showed a positive correlation with the 1st PCoA axis as well and contributed to the specificity of cabbage blends. (*E*)-β-ocimene (14) and α-zingiberene (44) were absent only in the blends obtained from cabbage and in addition with (*Z*)-β-elemene (34), β-elemene (35), α-acoradiene (40), (Z)-γ-bisabolene (47) and some other compounds, which showed that the negative correlation accounted for the separation of cabbage specimens in the PCoA plot (Figure 1b).

**Figure 2:**
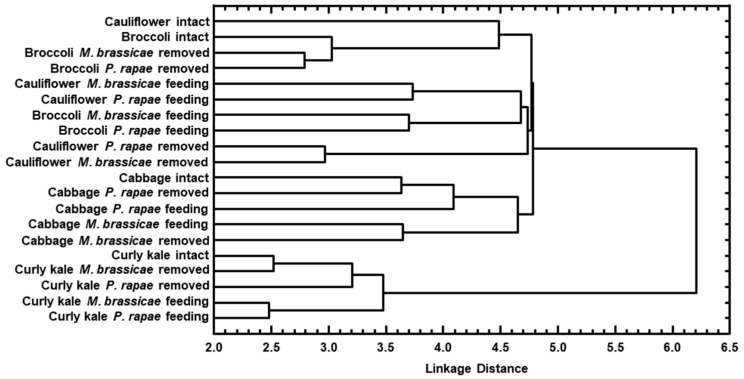
Dendrogram obtained by cluster analysis based on Euclidian distance between groups of four *Brassica oleraceae* cultivars of intact, infested with caterpillars of either *Mamestra brassicae* or *Pieris rapae* species and post-damaged stages.

Cluster analysis of odour bouquets revealed that the most distinct volatile blends originated from curly kale, followed by cabbage, and the most similar ones were obtained from cauliflower and broccoli (Figure 2).

### 2.3. Variation in the Odour Profiles of Plant-Herbivore Systems During Caterpillar Feeding

In total, 26, 45, 32 and 34 volatiles were detected from plants of cabbage, cauliflower, curly kale and broccoli, respectively, when caterpillars of *M. brassicae* were allowed to feed on the leaves for 24 h, while the damage caused by caterpillars of *P. rapae* resulted in the release of 28, 46, 32 and 26 volatiles, respectively (Table 1).

In the PCoA plot, the first two PCoA axes accounted for 29% and 17% of the total variation (Figure 1c). Eleven induced esters mainly composed of 4, 6 and 8 carbon chain length alcohol units and 4–6 carbon chain length acid units, including 20, 21, 24, 27, 28, 29, 30, 33, 36, 37, 49, together with nine sesquiterpenes, namely (*Z*)-β-elemene (34), β-elemene (35) (*E*)-β-farnesene (38), α-acoradiene (40), α-curcumene (41), β-selinene (42), α-zingiberene (44), (*Z*)-γ-bisabolene (47), (*E*)-α-bisabolene (48) and homoterpene (3*E*,7*E*)-4,8,12-trimethyltrideca-1,3,5,7,11-tetraene (50), positively contributed to the first PCoA axis and defined the specificity of the blends released by damaged plants of cauliflower (Figure 1c,d). Low proportions or the absence of the volatiles just mentioned as well as a significantly higher ratio of (*E*)-4,8-dimethylnona-1.3.7-triene (19) and unidentified (2, 45 and 52) in the blends of cabbage compared to those of other genotypes contributed to the specificity of the odour profile and separated specimens of cabbage from those of cauliflower, curly kale and partly from broccoli on the first PCoA axis (Figure 1c,d).

Methyl nerolate (31) was exclusively released from the damaged plants of curly kale. In addition, β-selinene (42), (*Z*)-β-elemene, (34), β-elemene (35), α−acoradiene (40) and β-myrcene (7) contributed to the specificity of the odour profile (Table 1, Figure 1c,d).

Inducible green leaf volatile (*Z*)-3-hexenyl acetate (8), two sesquiterpenes (*Z*,*E*)-α-farnesene (43) and (*E*,*E*)-α-farnesene (46), 2-methylbutyl isothiocyanate (12), β-cyclocitral (26), β-ionone (39) as well as three sulphides including dimethyl disulphide (1), dimethyl trisulphide (5) and dimethyl tetrasulphide (25) positively correlated with the second PCoA axis, and distinguished odour profiles of all varieties of plants released during caterpillar feeding from those originated from damaged plants after caterpillars were removed. In the PCoA plot, the stronger separation effect according to the second PCoA axis was observed when plants were damaged by caterpillars of *M. brassicae* compared to those of *P. rapae* species. The cluster of the compounds mentioned had the highest contribution to the odour bouquets released from broccoli plants, and the lowest impact was observed on the scent of curly kale (Figure 1c,d).

A comparison of odour bouquets by cluster analysis revealed that odour profiles obtained from cauliflower and broccoli were the most similar, followed by cabbage, and the most distinct were those released by curly kale. The order of similarity between cultivars was independent of which herbivore species caused damage (Figure 2).

### 2.4. Variation in the Odour Profiles of Damaged Plants After the Removal of Caterpillars and Faeces

After the caterpillars and faeces of *M. brassicae* were removed from the leaves, the volatile bouquets of cabbage, cauliflower, curly kale and broccoli were comprised of 24, 43, 31 and 28 volatiles, respectively, while after the removal of caterpillars and faeces of *P. rapae*, the damaged plants released 29, 43, 30 and 26 compounds, respectively (Table 1).

According to the first PCoA axis, the dissimilarity of the scores of odour bouquets obtained from the damaged plants with and without caterpillars indicated that most of the volatiles, which contributed to this PCoA axis, determined the specificity of odour profiles from damaged leaves in a similar manner as those from post-damaged leaves in the specimens of cauliflower, curly kale, cabbage and at the less extent in broccoli. For example, cauliflower plants continued to release induced esters (20, 21, 24, 27, 30, 33, 36, 37, 49) as well as sesquiterpenes (34, 35, 38, 40, 42, 44, 47, 48) and homoterpene TMTT (50) even after removal of caterpillars (Figure 1c,d).

The absence of sulphides in odour bouquets released from plants of all varieties, with exceptions of dimethyl trisulphide (5) in damaged plants by *P. rapae* and decrease in proportions of (*Z*)-3-hexenyl acetate (8), diminish the differences of placement of odour sets in respect to the second PCoA axis for cabbage, broccoli and to some extent for cauliflower plants (Figure 1c).

The exclusive presence of (*Z*)-methyl geranate (31) and high amounts of β-myrcene (7), limonene (11), elemenes (34, 35), α-acoradiene (40) and β-selinene in the sets of volatiles released from plants of the curly kale as well as the absence of (*Z*,*E*)-α-farnesene (43) (Table 1) contributed to the specificity of odour profiles and determine the placement of curly kale plants in the lower part of PCoA plot (Figure 1c,d).

The cluster analysis indicated that the most similar volatile blends released by post-damaged plants were recorded from cauliflower, broccoli and cabbage. The most distinct one was obtained from curly kale. The order of similarity between cultivars was independent of which herbivore species were previously feeding on the plant (Figure 2).

### 2.5. Variation in the Odour Profiles of M. brassicae Versus P. rapae Infested Plants

In the cabbage-herbivore systems, β-ionone (39) was induced at large amounts during *M. brassicae* feeding compared with the trace amounts emitted from the cabbage fed on by *P. rapae* larvae. (*Z*)-4-hexen-1-yl butanoate (21), *p*-menthan-3-one (22) and unidentified hydrocarbon (51) were exclusively released, and methyl-2-ethyl hexanoate (13), 1-methoxy-1-methylethyl-benzene (17), unidentified (23) and (Z)-3-hexen-1-yl 3-methylbutanoate (28) were sampled at larger amounts during feeding of *P. rapae* compared to the trace amounts released during feeding of *M. brassicae* (Table 1).

In the *M. brassicae* caterpillar-cauliflower plant system, 2-methylbutyl isothiocyanate (12), dimethyl tetrasulphide (25), hexyl 3-methylbutanoate (29) and 3-methyl-2-buten-1-yl hexanoate (30) were exclusively detected and (4, 5, 14-16, 26, 36, 39, 41, 42 and 49) were trapped in larger amounts compared to those trapped from the plants bearing *P. rapae* caterpillars. Whereas only unidentified (32) was explicitly released as well as (*E*)-α-bisabolene (48) occurred at larger amounts in the headspace of cauliflower plants fed on by *P. rapae* larvae (Table 1).

Odour bouquets of curly kale plants infested by *M. brassicae* caterpillars were characterised by the exclusive presence of dimethyl disulphide (1), 2-methylbutyl isothiocyanate (12) and (*E*)-β-ocimene (14) as well as by larger amounts of (4, 5, 18, 25, 26, 39, 43 and 46), compared to those released by *P. rapae* damaged plants that instead specifically produced (*Z*)-3-hexen-1-yl hexanoate (Table 1).

The difference of volatile blends released from *M. brassicae* caterpillar damaged broccoli was determined by the exclusive presence of four terpenoids (9, 18, 22, 50), two esters (24, 30), dimethyl tetrasulphide (25), 2-methylbutyl isothiocyanate (12) and 1-methoxy-1-methylethyl-benzene (17) as well as by larger amounts of (4, 5, 14, 16, 19, 26, 39, 44 and 52), compare to those released by the plants infested with *P. rapae* (Table 1).

The cluster analysis revealed that the most pronounced differences between the odour profiles of the same genotype when either of two herbivore species were feeding was registered for cabbage (Euclidean distance (ED) equals 4.6) followed by broccoli and cauliflower (ED’s were registered around 3.7) and the least pronounced differences were recorded for curly kale (ED = 2.5) (Figure 2).

### 2.6. Variation in the Odour Profiles of M. brassicae Versus P. rapae Damaged Plants After Removal of Caterpillars and Faeces

*p*-Menthan-3-one (22) and β-ionone (39) were exclusively present in the volatile blends of cabbage after *M. brassicae* caterpillars were removed compared to those obtained from post-damaged plants by *P. rapae* larvae, while *p*-mentha-2,4(8)-diene (18), (*Z*)-4-hexen-1-yl butanoate (21) and (*Z*)-3-hexen-1-yl 3-methylbutanoate (28) were exclusively released as well as γ-terpinene(15) and (*E*)-sabinene hydrate (16) occurred at larger amounts in odour bouquets released from post-damaged plants by *P. rapae* compare to those collected from cabbage previously bearing *M. brassicae* larvae (Table 1).

Unidentified (32) and β-ionone (39) were exclusively trapped, and eight volatiles (14-16, 26, 29, 36, 49 and 50) occurred in larger amounts from *M. brassicae* post-damaged cauliflower plants compared to plants after removing *P. rapae* larvae. Dimethyl trisulphide (5) and 3-methyl-2-buten-1-yl hexanoate (30) were exclusively detected in trace amounts from *P. rapae* larvae post-damaged cauliflower plants (Table 1).

Odour bouquets of curly kale plants previously infested by *M. brassicae* caterpillars were exclusively comprised of (8, 14, 29, 39, 43, 47, 48 and 52), as well as bore larger amounts of (3, 4, 6, 16, 46 and 47) compared to those obtained from plants after being damaged by *P. rapae*. In comparison, previous feeding of *P. rapae* caterpillars resulted in the exclusive production of dimethyl trisulphide (5) and larger amounts of (17, 27 and 28) compared to those obtained from plants post-damage by *M. brassicae* caterpillars (Table 1).

*p*-Mentha-2,4(8)-diene (18) and p-menthan-3-one (22) were exclusively present and (3, 4, 6, 7, 9, 14, 19, 39, 44 and 45) were found at large amounts in the volatile blends of broccoli specimens after *M. brassicae* caterpillars were removed compared to those obtained from post-damaged plants by *P. rapae* larvae.

The cluster analysis showed that the most pronounced differences in odour profiles of plants from the same genotype at the post-damage stage, caused by *M. brassicae* compared to *P. rapae* caterpillars, were observed in cabbage specimens (Euclidean distance (ED) = 4.6), followed by cauliflower (ED = 3.0), broccoli (ED = 2.8) and curly kale (ED = 2.5) (Figure 2).

Moreover, the cluster analysis showed that in the curly kale, broccoli and cauliflower, the differences in odour bouquets were more expressed between plants with and without active feeding, independent of the herbivore identity, while in the cabbage, the larger differences were observed between odour profiles with different herbivores feeding, independent of if the herbivore was present or removed (Figure 2).

## 3. Discussion

The grouping of compounds in the odour samples was determined by plant genotype rather than by the attack of herbivores. This fact showed that plant genotype affects the composition of the volatile blends more than herbivores do. The odour bouquets of intact *B. oleracea* plants predominantly comprised of various terpenoids, including monoterpenes, sesquiterpenes and their derivatives as well as homoterpenes. The sesquiterpenes, (*Z*)-β-elemene (34), β-elemene (35), α-acoradiene (40), β-selinene (42) contributed to the specificity of the curly kale odour (Table 1). The strong correlation among these sesquiterpenes may reflect shared biosynthetic pathways. According to our knowledge, these compounds, except β-elemene, have not been previously reported to occur in the headspace of *B. oleracea* genotypes. Two monoterpenes, β-myrcene (7) and limonene (11), were determined at relatively high amounts in the curly kale blends and these volatiles are present in four genotypes we have analysed as well as in other crucifers and many other plant species [24]. In our experiments, odour profiles obtained from the headspace of the healthy plants contained small amounts of green leaf volatile (*Z*)-3-hexenyl acetate (8) except in cauliflower, where this acetate was not detected. The general similarity of the current results and those for other intact crucifer varieties [25,26,27,28,29] indicated that intact plants we have used for experiments were under low, if any, stress levels while extremely high amounts (97%) of (*Z*)-3-hexenyl acetate reported from the headspace of intact curly kale variety *acephala* [30] occur most probable due to some specificity of the genotype or an unidentified stress. Therefore, curly kale had the most distinctive odour profiles from those of the other three genotypes (Figure 2).

Cauliflower and broccoli had the most similar odour profiles, predominantly determined by (*E*,*E*)-α-farnesene (46). Plants of curly kale and cabbage released much lower amounts of this sesquiterpene. Our data aligned well with findings that several cabbage genotypes released low amounts of (*E*,*E*)-α-farnesene [26,28] where the absence of this sesquiterpene was reported by Geervliet et al. [25], Conti et al. [27] and Shiojiri et al. [29]. (*E*,*E*)-α-farnesene was also absent in odour bouquets of curly kale [30].

Current data showed that esters and terpenes were most affected by the feeding of the two herbivore species tested here. (*Z*)-3-hexenyl acetate (8) alone accounted for a quarter to one-third of total volatile emission in all genotypes. This green leaf volatile is produced from the oxidative degradation of plant lipids when enzymes are liberated after plant tissue damage [31], and it is common in the odours of many plant species under herbivore attack [32]. In addition, it has been extensively described in the literature as important in affecting insect-plant interactions at different trophic levels [31,33]. Ten esters were induced exclusively or at larger amounts in the cauliflower compared to the specimens of other genotypes and contributed to the specificity of the cauliflower blends. To our knowledge, the presence of most of these esters in the volatile mixtures of cauliflower is reported for the first time, and olfactory activities, as well as a behavioural function of the compounds to *M. brassicae* and *P. rape*, are not known. Smid et al. have found that (Z)-3-hexen-1-yl butanoate (24), (Z)-3-hexen-1-yl 2-methylbutanoate (27) and (Z)-3-hexen-1-yl 3-methylbutanoate (28) elicited electroantennographic responses in two parasitoid species of *P. rapae* while their behavioural function is unknown [34] (Table 2). Methyl nerolate (31) was released explicitly by curly kale with herbivores present as well as after caterpillars and faeces were removed and contributed to the specificity of odour blends of that genotype.

The second most abundant compound (*E*,*E*)-α-farnesene (46), together with other two isomers (Z,*E*)-α-farnesene (43) and (*E*)-β-farnesene (38), were induced by feeding of caterpillars and contributed to the specificity of cauliflower as well as broccoli odours. The (*E*)-β-farnesene exclusively appeared only in cauliflower odours. An increase in production of (*E*,*E*)-α-farnesene in response to herbivore was reported in two of three cabbage genotypes [28], and the treatment of cabbage genotypes by methyl jasmonate increased production by around ten times compared with the control [26].

The plants of the family Brassicaceae are known to be rich in sulphur compounds including isothiocyanates and sulphides [3,24]. In our experiments, four sulphur-containing compounds, i.e., methylbutyl isothiocyanate (12), dimethyl disulphide (1), dimethyl trisulphide (5) and dimethyl tetrasulphide (25), were present predominantly in the volatile bouquets obtained from herbivore-plant systems. In intact plants, glucosinolates and the enzyme myrosinase are spatially segregated [35], but tissue damage brings them together, which facilitates glucosinolate hydrolysis into thiocyanates, isothiocyanates, nitriles, oxazolidine-2-thiones and epithionitriles, depending upon the structure of glucosinolates, pH and other conditions [36]. These are putatively described as defensive compounds and provide protection against many generalist herbivores. It was reported that several isothiocyanates demonstrate olfactory activity in females of both *M. brassicae* and *P. rapae* [37,38] as well as mediating diverse interactions at different tropic levels [39], unfortunately, olfactory and behavioural activities of 2-methylbutyl isothiocyanate are not well described. We have found that dimethyl trisulphide (5) was present in the volatile sets collected from intact curly kale plants at low amounts, and it has been reported to occur in the volatile blends of intact kale [30] as well as of intact cabbage genotypes [25]. In addition, sulphides, to a large extent, are released from the faeces of larvae [17,39] and could be produced by microorganisms inhabiting the faeces [40]; thus, the source of their release could be plants, faeces and microbes.

The grouping of odour samples in the clusters in the PCoA plots was determined by genotype type rather than plant status. This fact showed that plant genotype affects the composition of the volatile blends more than herbivores do. Similar data are known for apple trees and spider mites system [40], corn, cotton and Lepidoptera larvae [41], cabbage varieties and *Pieris* caterpillars [25]. The genotype-driven variation of odour profiles makes those blends more consistent and predictable under various herbivore feeding. This consistency allows for the development of genotypes with specific VOC profiles that can deter or decrease plants’ attractiveness to pests as well as attract and maintain a stable population of natural enemies. By selecting cultivars with specific VOC profiles, a contribution can be made to an Integrated Pest Management strategy that allows farmers to reduce their reliance on chemical pesticides and promote sustainable agriculture.

Our study revealed that the qualitative and quantitative differences in odour profiles between four genotypes of *Brassica oleracea* were sufficiently large to be detected by multivariate analysis techniques. However, the key question is whether insects are able to use those volatiles to detect a preferred genotype? Published data dealing with olfactory active volatiles showed that seven and four terpenoids evoked electrophysiological responses in the antennae of *M. brassicae* and *P. rapae* females, respectively, however, only α-terpinene and 1,8-cineole elicited upwind flight in *M. brassicae* (Table 2). PCoA analyses indicated that olfactory active compounds present in the odour sets of intact genotypes could provide some information for *M. brassicae* species about the identity of a genotype (Figure 1). For a complete picture, additional experiments are needed to determine whether other volatiles elicit electrophysiological and behavioural responses in *M. brassicae* and *P. rapae* are and play a significant role in distinguishing between genotypes.

**Table 2:** List of compounds reported as olfactory and/or behaviourally active for *Mamestra brassicae*, *Pieris rapae* females and their caterpillar parasites and were identified from four *B. oleracea* genotypes.

Compound Name	*M. brassicae*		*P. rapae*		*C. gl*	*C. ru*	*M. me*
	OA	BA	OA	BA	OA	OA	OA
Monoterpenes							
α-Terpinene	[42] ^1^	UF [42]					
γ-Terpinene	[37]		[43]				
β-Myrcene			[38,44]				
Limonene			[38,44]		[34]	[34]	
α-Pinene			[38]				
Oxygenated monoterpenes							
1.8-Cineole	[37,42]	UF [42]			[45]		
Sesquiterpenes							
(*E*,*E*)-α-Farnesene	[37]						
(*E*)-β-Farnesene	[37]						
(*Z*,*E*)-α-Farnesene	[37]						
Homoterpenes							
TMTT	[37]						
(*E*)-4,8-Dimethylnona-1,3,7-triene					[34]	[34]	[46]
Ketones							
β-Ionone			[38]	D [47]	[45]		
Esters							
(*Z*)-3-Hexen-1-yl acetate	[37,42]	UF; L [42]	[38]		[34,45]	[34]	[46]
(*Z*)-3-Hexenyl butanoate					[34,45]	[34]	
(*Z*)-3-Hexenyl 2-methylbutanoate					[34,45]	[34]	
(Z)-3-Hexenyl 3-methylbutanoate					[34,45]	[34]	

*C. gl*—*Cotesia glomerata*; *C. ru*—*Cotesia rubecula*; *M. me*—*Microplitis mediator*; OA—olfactory active determined by electroantennographic recording and/or single-cell recording; BA—behaviourally active; UF—upwind flight; L—landing; D—deterrent; TMTT—(3*E*,7*E*)-4,8,12-trimethyltrideca-1,3,5,7,11-tetraene; ^1^ Reference.

The damage caused by both Lepidoptera herbivore species increased the specificity of odour bouquets and made them more distinct. It remains to be tested whether the increased differences in volatile blends enhance the ability of herbivores to detect preferred genotypes. The attractiveness of intact versus damaged plant bouquets to herbivores could be increased or decreased [26,48,49,50] depending on several factors like the specificity of herbivore species, the extent of damage caused to plants, or the tissue damaged [32]. It was reported that induced volatiles (*Z*)-3-hexen-1-yl acetate (8), (*E*)-β-farnesene (38), (Z,*E*)-α-farnesene (43), (*E*,*E*)-α-farnesene (46) and TMTT (50) were olfactory active and (*Z*)-3-hexen-1-yl acetate mediated upwind flight and landing in *M. brassicae* females (Table 2), consequently, for cabbage moths, odour bouquets of damaged plants could increase attractiveness. It is known for some plant-herbivore systems that polyphagous herbivores, like *M. brassicae,* are attracted to damaged plants and increase the attack rate on these plants [32]. Contrary to polyphagous, specialist herbivores usually avoid plants releasing high levels of induced VOCs. β-Ionone, exclusively released by damaged plants, elicited olfactory responses in *Pieris rapae* butterflies and functioned as an oviposition deterrent (Table 2).

Moreover, *Brassica* phytochemical volatiles are essential for the effective location of herbivores by their predators and parasites, thereby reducing the extent of herbivory that plants experience [34,45,51,52]. However, the behaviour-modifying effect of single VOCs, as well as the composition of the blends effectively attracting parasites and parasitoids, remains unknown. Continued work is required to more fully understand the role played by VOCs in the highly dynamic *Brassica*-herbivore-predator/parasite systems with respect to genotypes emitting disfavoured volatile blends for herbivores and the preferable bouquets for enemies of herbivores. Increasing our understanding of the dynamics of these complex systems can contribute to the development of more sustainable pest management systems.

## 4. Materials and Methods

### 4.1. Plants and Insects

Plants of cabbage *Brassica oleracea* L. subs. *capitata*, cv. Consul, cauliflower *B. oleracea* L. subs. *botrytis*, cv. Nautilus, curly kale *B. oleracea* L. subs. *acephala*, cv. Vinterbo and broccoli *B. oleracea* L. subs. *italica*, cv. Marathon were grown from the seeds in the greenhouse of the Department of Ecology, SLU, Uppsala in 10 × 10 cm pots, light:dark 18:6. At the 5–6 true-leaf growth stage, plants were transported to the Royal Institute of Technology, Stockholm and were allowed to adapt for one week under laboratory conditions. Photoperiod consisted of 18:6 h light:dark phases. Two 400 W metal halide lamps (Philips HPI-T Plus, Amsterdam, The Netherlands) were used as the light source. The temperature was kept at 24 °C ± 2 °C during the photophase and 20 °C ± 2 °C during the scotophase.

Cabbage moth, *Mamestra brassicae* (L.) and small white, *Pieris rapae* L. were based on insects kindly supplied by the University of Wageningen, Department of Entomology.

After hatching, caterpillars were transformed to the Royal Institute of Technology and were held under the same conditions as the plants. Larvae were divided into four groups and fed on plants of the same genotypes as those used during the volatile collection experiments.

### 4.2. Sampling of Volatiles

We focused our attention on volatile phytochemical cues. Therefore, visual differences among *B. oleracea* genotypes were minimised by the use of small plants at the 5–6 true-leaf growth stage for odour sampling as well as for bioassay test. The Solid Phase Micro-Extraction (SPME) technique [53] was used to collect volatiles released from individual potted plants, bearing 6–7 leaves. The aboveground part of the plant was placed into a 1.4 L glass bell jar with top and side tubular openings and ground flange. At the bottom, the jar was closed by folding plates covered with aluminium foil to prevent soil odours from entering the jar. Half an hour before odour collection, filtered humidified air was pumped by universal sample pump PCXR8 (SKC Inc., Eighty Four, PA, USA) through the jar bearing plant at the flow rate 0.3 L/min. After pumping, both tubular openings of the jar were closed by aluminium foil. The routine purification of the SPME fibre, 100 mm polydimethylsiloxane (Supelco, Sigma-Aldrich group, Bellefonte, PA, USA) was done at 225 °C for about 10 min in a GC injector before each odour sampling. The syringe needle was then placed in the jar through the top tubular opening, and the purified fibre covered with adsorbent was exposed to a headspace. The collection lasted for 22 h, and it was carried out under the same laboratory conditions as described in the subchapter *Plants and insects*. Extended sampling periods of VOCs were selected to ensure that less abundant or temporally variable compounds would be captured, providing a more representative sample of the plant’s overall volatile emissions [54]. Volatiles were sampled from individual intact and post-wounded plants as well as from the plants during caterpillar feeding.

Insect feeding damage to the plants was caused by six *M. brassicae* or *P. rapae* caterpillars of about 1.5 cm and 1 cm body length, respectively, feeding on each plant during odour collection periods. Afterwards, caterpillars and faeces were removed from a plant, and the glass jar was replaced with a clean one. Two hours after the removal of caterpillars, volatiles from the post-damaged plants were collected for 22 h.

### 4.3. Analysis of Volatiles

Volatiles were analysed by means of a Varian 3400 gas chromatograph (GC), connected to a Finnigan SSQ 7000 mass spectrometer (MS) (ThermoFisher Scientific, Waltham, MA, USA). A SPB-1 silica capillary column (30 m, i.d. 0.25 mm, film thickness 0.25 μm; Supelco, Sigma-Aldrich group, Bellefonte, PA, USA) was used with a temperature program of 40 °C (1 min), increased by 4 °C/min to 200 °C, then by 10 °C/min up to 230 °C and thereafter held isothermally at 230 °C for 1 min. The split/splitless injector temperature was 225 °C, and the splitless period lasted for 60 s. Helium was used as the carrier gas, with an inlet pressure of 70 kPa. Electron ionisation mass spectra were determined at 70 eV with an ion source at 150 °C. Volatiles were identified by comparison of their mass spectral data and GC retention times with the corresponding data available from NIST version 2.0 mass spectral search programme (National Institute of Standards and Technology, Gaithersburg, MD, USA) and MassFinder 3 mass spectral and retention indexes database (Dr. Hochmuth scientific consulting, Hamburg, Germany) as well as those of a synthetic standard for the compounds indicated in Table 1.

### 4.4. Statistical Analyses

To assess and visualise the associations between cases, i.e., odour bouquets of brassica genotypes, and variables, i.e., volatile compounds, the principal coordinate analyses (PCoA) were performed on log-transformed absolute amounts of volatiles using the software Canoco Version 4.54 (Ter Braak and Smilauer, Biometris Plant Research International, Amsterdam, The Netherlands).

Cluster analysis of odour bouquets was carried out to reveal a degree of difference between genotypes as well as between the plants of different statuses using Statistica 6.0 software (StatSoft, Tulsa, OK, USA).

To determine the significant differences between the amounts of volatiles released by genotypes within appropriate plant status, the data were log-transformed and analysed by nonparametric Conover–Iman test with Bonferroni corrections (Table 1) using software R, version 4.0.2 and Rstudio, version 1.3.959 (R Foundation for Statistical Computing, Institute for Statistics and Mathematics, Wirtschaftsuniversität Wien, Vienna, Austria).

## 5. Conclusions

This study demonstrated that (1) qualitative and quantitative differences in odour profiles between four genotypes of *B. oleracea* were sufficient to differentiate between groups using multivariate analysis techniques; (2) plant genotypes affected the composition of the volatile blends at the large extent compare to two herbivore species tested; (3) the most distinct volatile blends were originated from curly kale followed by cabbage, cauliflower and broccoli; (4) most pronounced differences between the odour profiles of the same genotype when either of two herbivore species were feeding has been registered for cabbage followed by broccoli, cauliflower and curly kale; (5) in the curly kale, broccoli and cauliflower the differences of odour bouquets were more expressed between plants with and without active feeding, independent of the herbivore identity, while in the cabbage the larger differences were observed between odour profiles with different herbivores feeding, independent of if the herbivore was present or removed. The importance of genotype-driven variation in odour profiles lies in its consistency, predictability and potential for integration into breeding programs and IPM strategies. By focusing on the genetic basis of VOC emissions, researchers and breeders can develop more effective and sustainable plant protection methods that leverage the natural defence mechanisms of plants.

## Figures and Tables

**Table 1:** The composition of odour blends originated from the headspace of four *Brassica oleraceae* cultivars of intact, infested with caterpillars of either *Mamestra brassicae* or *Pieris rapae* species and from post-damaged plant stages.

No	Compound	CAS No	Group	RI	ID	Intact				*M. brassicae* Caterpillars Feeding			
						CB	CL	CK	BR	CB	CL	CK	BR
1	Dimethyl disulphide	624-32-0	S	-	RC	nd	nd	nd	nd	tr	5.5 ± 0.4 a	5.3 ± 0.4 a	5.8 ± 0.5 a
2	Unidentified (oxime)		IM	908	U	6.4 ± 0.1 a	5.4 ± 0.2 c	6.3 ± 0.1 ab	6.1 ± 0.1 b	6.7 ± 0.3 a	5.6 ± 0.5 b	5.9 ± 0.0 *b	6.3 ± 0.2 a
3	α-Thujene	2867-05-2	MT	923	RC	5.6 ± 0.1 a	5.3 ± 0.4 a	6.1 ± 0.3 a	5.6 ± 0.1 a	5.2 ± 0.3 a	4.9 ± 0.4 a	5.1 ± 0.5 a	4.7 ± 0.5 a
4	α-Pinene	80-56-8	MT	930	RC	4.9 ± 0.3 a	4.1 ± 1.1 a	4.3 ± 0.3 a	tr	4.8 ± 0.2 b	3.7 ± 1.0d	5.6 ± 0.0 *a	4.2 ± 0.3 c
5	Dimethyl trisulphide	3658-80-8	S	944	RC	nd	nd	tr	nd	nd	5.5 ± 0.5 a	5.2 ± 0.4 a	5.8 ± 0.5 a
6	Sabinene	3387-41-5	MT	966	RC	6.4 ± 0.3 ab	6.0 ± 0.3 b	6.6 ± 0.1 a	6.2 ± 0.2 ab	6.2 ± 0.1 b	6.1 ± 0.2 b	6.8 ± 0.1 a	5.9 ± 0.3 b
7	β-Myrcene	123-35-3	MT	985	RC	6.5 ± 0.5 b	6.5 ± 0.2 b	7.1 ± 0.1 a	6.6 ± 0.1 b	6.5 ± 0.2 b	6.7 ± 0.1 b	7.3 ± 0.1 a	6.5 ± 0.2 b
8	(Z)-3-Hexen-1-yl acetate	3681-71-8	E	991	RC	6.0 ± 0.2 a	nd	6.1 ± 0.0 *a	4.0 ± 1.1 b	6.6 ± 0.2 c	7.2 ± 0.2 b	7.6 ± 0.1 a	7.0 ± 0.1 b
9	α-Terpinene	99-86-5	MT	1008	RC	3.8 ± 0.0 *	tr	nd	nd	tr	tr	nd	tr
10	1,8-Cineole	470-82-6	OMT	1019	RC	6.1 ± 0.1 a	6.3 ± 0.3 a	6.1 ± 0.1 a	5.8 ± 0.1 b	5.8 ± 0.3 ab	6.0 ± 0.1 a	6.1 ± 0.1 a	5.6 ± 0.1 b
11	Limonene	7705-14-8	MT	1021	RC	6.5 ± 0.0 *b	6.5 ± 0.2 b	7.2 ± 0.1 a	6.6 ± 0.1 b	6.6 ± 0.1 b	6.7 ± 0.1 b	7.2 ± 0.1 a	6.6 ± 0.1 b
12	2-Methylbutyl isothiocyanate	4404-51-7	IST	1029	MS	nd	nd	1.3 ± 1.3	nd	nd	2.9 ± 1.2 a	tr	3.4 ± 0.9 a
13	Methyl-2-ethyl hexanoate	816-19-3	E	1033	MS	tr	5.0 ± 0.3 b	tr	5.7 ± 0.1 a	tr	4.7 ± 0.4 b	5.1 ± 0.3 ab	5.5 ± 0.2 a
14	(*E*)-β-Ocimene	3779-61-1	MT	1042	RC	nd	tr	tr	4.4 ± 0.3	tr	3.9 ± 1.0 a	4.1 ± 0.2 a	4.2 ± 0.3 a
15	γ-Terpinene	99-85-4	MT	1051	RC	4.7 ± 0.4 b	tr	5.3 ± 0.4 ab	5.3 ± 0.1 a	3.8 ± 0.9	3.3 ± 0.9 b	5.5 ± 0.1 a	5.1 ± 0.3 a
16	(*E*)-Sabinene hydrate	17699-16-0	OMT	1056	RC	5.2 ± 0.3 ab	tr	5.5 ± 0.1 a	4.9 ± 0.3 b	3.9 ± 0.9 b	5.1 ± 0.3 b	5.7 ± 0.1 a	4.9 ± 0.3 b
17	1-Methoxy-1-methylethyl-benzene	935-67-1	AR	1075	MS	4.5 ± 0.4 a	3.5 ± 1.0 b	tr	5.0 ± 0.3 a	tr	4.1 ± 0.3	tr	tr
18	*p*-Mentha-2,4(8)-diene	586-63-0	MT	1078	RC	5.0 ± 0.3 a	tr	4.9 ± 0.3 a	4.4 ± 0.3 a	4.6 ± 0.3 b	3.8 ± 1.0 c	5.4 ± 0.0 *a	4.1 ± 0.3 bc
19	(*E*)-4,8-Dimethylnona-1,3,7-triene	19945-61-0	HT	1107	MSRI	5.5 ± 0.1	nd	nd	tr	5.9 ± 0.2 a	1.1 ± 1.1 c	nd	5.2 ± 0.3 b
20	2-Methylbutyl valerate	55590-83-5	E	1129	RC	nd	nd	nd	nd	nd	5.1 ± 0.5	nd	nd
21	(Z)-4-Hexen-1-yl butanoate	69727-41-9	E	1131	MS	nd	tr	nd	nd	nd	5.2 ± 0.6	nd	nd
22	*p*-Menthan-3-one	10458-14-7	OMT	1132	RC	nd	nd	nd	nd	nd	2.2 ± 1.4 c	4.6 ± 0.3 a	4.0 ± 1.0 b
23	Unidentified			1152	MS	5.4 ± 0.1 b	5.6 ± 0.0 *a	tr	nd	5.5 ± 0.1 b	5.6 ± 0.1 ab	5.7 ± 0.0 *a	4.8 ± 0.4 c
24	(Z)-3-Hexen-1-yl butanoate	16491-36-4	E	1171	RC	nd	nd	nd	nd	nd	6.8 ± 0.3 a	nd	4.3 ± 1.1 b
25	Dimethyl tetrasulphide	5756-24-1	S	1181	RC	nd	nd	nd	nd	nd	3.4 ± 1.4 b	3.3 ± 0.8 b	5.3 ± 0.4 a
26	β-Cyclocitral	432-25-7	OMT	1194	RC	nd	nd	nd	nd	tr	4.8 ± 0.4 a	4.7 ± 0.4 a	5.4 ± 0.4 a
27	(Z)-3-Hexen-1-yl 2-methylbutanoate	53398-85-9	E	1217	RC	nd	nd	nd	nd	5.7 ± 0.0 *c	6.8 ± 0.3 a	6.1 ± 0.1 b	5.8 ± 0.2 bc
28	(Z)-3-Hexen-1-yl 3-methylbutanoate	35154-45-1	E	1220	RC	nd	nd	nd	nd	tr	6.9 ± 0.3 a	5.7 ± 0.1 b	5.5 ± 0.2 b
29	Hexyl 3-methylbutanoate	10632-13-0	E	1228	RC	nd	nd	nd	nd	nd	3.4 ± 0.9	nd	nd
30	3-Methyl-2-buten-1-yl hexanoate	74298-89-8	E	1231	MS	nd	nd	nd	nd	nd	5.0 ± 0.5	nd	tr
31	Methyl nerolate	1862-61-9	E	1263	MSRI	nd	nd	nd	nd	nd	nd	5.5 ± 0.1	nd
32	Unidentified			1315	U	nd	nd	nd	nd	nd	nd	nd	nd
33	(Z)-3-hexen-1-yl hexanoate	31501-11-8	E	1324	RC	nd	nd	nd	nd	nd	6.3 ± 0.4	nd	nd
34	(Z)-β-Elemene	674819-48-8	ST	1376	MSRI	nd	tr	6.0 ± 0.0 *	nd	nd	5.4 ± 0.1 b	6.0 ± 0.1 a	nd
35	β-Elemene	33880-83-0	ST	1383	RC	nd	6.3 ± 0.2 b	7.3 ± 0.0 *a	nd	nd	6.9 ± 0.2 b	7.4 ± 0.1 a	nd
36	Unidentified (ester)		E	1419	U	nd	nd	nd	nd	nd	4.5 ± 0.4	nd	nd
37	Octyl 3-methylbutanoate	7786-58-5	E	1424	MS	nd	nd	nd	nd	nd	4.1 ± 0.3	nd	nd
38	(*E*)-β-Farnesene	18794-84-8	ST	1447	RC	nd	nd	nd	nd	nd	5.1 ± 0.3	nd	nd
39	β-Ionone	79-77-6	KT	1461	RC	nd	nd	nd	nd	3.3 ± 0.8 b	4.9 ± 0.4 a	4.9 ± 0.3 a	5.4 ± 0.4 a
40	α-Acoradiene	87173-79-3	ST	1463	MSRI	nd	4.2 ± 0.3 b	5.8 ± 0.1 a	nd	nd	5.1 ± 0.3 b	5.7 ± 0.0 *a	nd
41	α-Curcumene	644-30-4	ST	1468	MSRI	nd	tr	nd	nd	nd	5.0 ± 0.3	nd	nd
42	β-Selinene	21488-94-8	ST	1474	MSRI	nd	nd	5.2 ± 0.3	nd	nd	3.3 ± 0.9 b	5.0 ± 0.3 a	nd
43	(Z,*E*)-α-Farnesene	26560-14-5	ST	1482	RC	nd	nd	nd	5.1 ± 0.3	tr	5.2 ± 0.4 a	3.3 ± 0.8 b	5.4 ± 0.2 a
44	α-Zingiberene	495-60-3	ST	1483	MSRI	nd	tr	5.7 ± 0.1 a	5.4 ± 0.0 *b	nd	5.9 ± 0.1 a	5.5 ± 0.1 b	5.6 ± 0.1 b
45	Unidentified			1488	U	6.5 ± 0.1 a	6.2 ± 0.0 *b	6.3 ± 0.1 b	5.9 ± 0.1 c	6.1 ± 0.0 *a	5.6 ± 0.5 ab	6.0 ± 0.0 *a	4.5 ± 1.1 b
46	(*E*,*E*)-α-Farnesene	502-61-4	ST	1495	RC	5.9 ± 0.1 b	6.9 ± 0.1 a	5.4 ± 0.1 c	7.1 ± 0.0.1 a	6.6 ± 0.1 b	7.1 ± 0.2 a	5.9 ± 0.1 c	7.2 ± 0.1 a
47	(Z)-γ-Bisabolene	495-62-5	ST	1502	MSRI	nd	tr	tr	nd	nd	5.0 ± 0.3 a	5.4 ± 0.4 a	tr
48	(*E*)-α-Bisabolene	17627-44-0	ST	1530	MSRI	nd	nd	nd	nd	nd	tr	nd	nd
49	(Z)-3-Hexen-1-yl benzoate	25152-85-6	E	1542	RC	nd	nd	nd	nd	nd	1.9 ± 1.2	nd	nd
50	TMTT *	62235-06-7	HT	1566	RC	nd	nd	nd	nd	nd	tr	nd	1.1 ± 1.1
51	Unidentified (branched hydrocarbon)		HY	1937	U	4.6 ± 1.2	nd	nd	nd	nd	nd	nd	nd
52	Unidentified (MS similar to verticilol)			1990	U	5.3 ± 1.3	nd	nd	nd	5.7 ± 0.2 a	nd	nd	4.1 ± 0.3 b
**No**	**Compound**	***M. brassicae*** **caterpillars removed**				***P. rapae*** **caterpillars feeding**				***P. rapae*** **caterpillars removed**			
		**CB**	**CL**	**CK**	**BR**	**CB**	**CL**	**CK**	**BR**	**CB**	**CL**	**CK**	**BR**
1	Dimethyl disulphide	nd	nd	nd	nd	tr	5.2 ± 0.7 a	nd	5.3 ± 0.7 a	nd	nd	nd	nd
2	Unidentified (oxime)	6.2 ± 0.2 a	6.0 ± 0.2 a	6.0 ± 0.2 a	6.1 ± 0.2 a	5.9 ± 0.1 b	6.3 ± 0.1 a	6.2 ± 0.2 a b	6.0 ± 0.0 *b	5.8 ± 0.1 b	6.2 ± 0.1 a	6.3 ± 0.2 a	5.9 ± 0.4 ab
3	α-Thujene	5.2 ± 0.1 b	5.3 ± 0.4 b	6.1 ± 0.1 a	5.4 ± 0.4 b	5.9 ± 0.1 a	5.4 ± 0.1 b	5.8 ± 0.1 a	4.8 ± 0.5 b	5.8 ± 0.1 a	5.3 ± 0.2 b	5.7 ± 0.1 a	5.1 ± 0.1 b
4	α-Pinene	4.8 ± 0.4 a	tr	5.4 ± 0.2 a	4.6 ± 0.5 a	5.3 ± 0.3	tr	tr	tr	4.9 ± 0.5	tr	tr	tr
5	Dimethyl trisulphide	nd	nd	nd	tr	nd	tr	tr	tr	nd	tr	4.9 ± 0.6	nd
6	Sabinene	6.4 ± 0.2 b	6.1 ± 0.2 b	6.9 ± 0.1 a	6.1 ± 0.4 b	6.5 ± 0.2 a	5.7 ± 0.2 b	6.3 ± 0.0 *a	5.8 ± 0.3 b	6.7 ± 0.2 a	5.5 ± 0.1 d	6.3 ± 0.0 *b	5.9 ± 0.0 * c
7	β-Myrcene	6.0 ± 0.1 c	6.8 ± 0.1 b	7.4 ± 0.1 a	6.7 ± 0.3 b	6.7 ± 0.1 b	6.7 ± 0.1 b	7.2 ± 0.1 a	6.5 ± 0.1 b	6.9 ± 0.2 a	6.8 ± 0.2 a b	7.1 ± 0.2 a	6.6 ± 0.0 *b
8	(Z)-3-Hexen-1-yl acetate	6.1 ± 0.4 a	6.3 ± 0.1 a	5.9 ± 0.3 a	6.1 ± 0.1 a	6.8 ± 0.6 a	7.3 ± 0.3 a	7.0 ± 0.5 a	6.7 ± 0.4 a	6.1 ± 0.1 a	5.2 ± 0.8 b	6.1 ± 0.5 a b	5.9 ± 0.0 *b
9	α-Terpinene	tr	tr	4.7 ± 0.4 a	2.9 ± 1.2 b	tr	tr	nd	nd	tr	tr	nd	nd
10	1,8-Cineole	5.8 ± 0.1 b	6.0 ± 0.1 b	6.3 ± 0.1 a	5.9 ± 0.2 b	6.3 ± 0.1 a	5.6 ± 0.1 c	5.9 ± 0.1 b	5.9 ± 0.0 *b	6.4 ± 0.1 a	5.6 ± 0.0 * c	6.0 ± 0.0 *b	6.2 ± 0.3 a b
11	Limonene	6.5 ± 0.1 b	6.8 ± 0.2 b	7.4 ± 0.1 a	6.6 ± 0.3 b	6.8 ± 0.1 a	6.4 ± 0.0 *b	7.0 ± 0.1 a	6.5 ± 0.1 b	6.9 ± 0.2 a	6.5 ± 0.1 c	7.0 ± 0.1 a	6.6 ± 0.0 *b c
12	2-Methylbutyl isothiocyanate	nd	nd	nd	nd	nd	nd	nd	nd	nd	nd	nd	nd
13	Methyl-2-ethyl hexanoate	5.2 ± 0.1 a	4.7 ± 0.4 a	5.1 ± 0.3 a	5.2 ± 0.4 a	4.7 ± 0.4 b	5.3 ± 0.2 b	4.8 ± 0.0.5 b	5.7 ± 0.0 *a	5.5 ± 0.2 b	5.6 ± 0.1 b	5.6 ± 0.0 *b	6.0 ± 0.0 *a
14	(*E*)-β-Ocimene	tr	4.2 ± 0.5 a	4.1 ± 0.3 a	4.0 ± 1.1 a	tr	tr	nd	tr	tr	tr	nd	4.2 ± 0.3
15	γ-Terpinene	tr	3.7 ± 1.0 b	5.4 ± 0.2 a	5.1 ± 0.4 a	5.4 ± 0.1 a	tr	5.5 ± 0.2 a	4.7 ± 0.5	5.1 ± 0.6 a b	tr	5.3 ± 0.0 *b	5.4 ± 0.0 *a
16	(*E*)-Sabinene hydrate	tr	4.8 ± 0.4 b	5.9 ± 0.0 *a	4.8 ± 0.4 b	5.8 ± 0.1 a	tr	5.7 ± 0.0 *b	tr	5.1 ± 0.7 a	tr	5.3 ± 0.1 a	5.3 ± 0.0 *a
17	1-Methoxy-1-methylethyl-benzene	tr	tr ±	4.4 ± 0.4	nd	4.2 ± 0.3 b	5.6 ± 0.1 a	tr	nd	tr	tr	5.7 ± 0.1	nd
18	*p*-Mentha-2,4(8)-diene	nd	nd	5.5 ± 0.1	tr	5.2 ± 0.1 a	5.2 ± 0.0 *a	tr	nd	4.4 ± 0.5 b	nd	5.2 ± 0.0 *a	nd
19	(*E*)-4,8-Dimethylnona-1,3,7-triene	6.0 ± 0.3 a	nd	nd	5.6 ± 0.3 a	5.7 ± 0.2 a	4.8 ± 0.5 b	nd	tr	2.0 ± 2.0	nd	nd	tr
20	2-Methylbutyl valerate	nd	4.5 ± 1.2	nd	nd	nd	5.1 ± 0.1	nd	nd	nd	4.9 ± 0.0 *	nd	nd
21	(Z)-4-Hexen-1-yl butanoate	nd	5.3 ± 0.1	nd	nd	5.5 ± 0.1 a	5.6 ± 0.2 a	nd	nd	1.8 ± 1.8 b	5.2 ± 0.3 a	nd	nd
22	*p*-Menthan-3-one	4.8 ± 0.4 a	5.2 ± 0.1 a	4.1 ± 0.2 b	tr	5.1 ± 0.1 b	5.3 ± 0.0 *a	4.9 ± 0.5 a b	nd	nd	5.0 ± 0.0 *a	4.8 ± 0.5 a	nd
23	Unidentified	5.2 ± 0.1 c	5.8 ± 0.2 a	5.4 ± 0.1 bc	5.6 ± 0.1 ab	5.7 ± 0.0 *b	5.9 ± 0.1 a	5.8 ± 0.1 a b	5.3 ± 0.2 c	5.5 ± 0.2 b	5.9 ± 0.1 a	5.9 ± 0.1 a	5.6 ± 0.0 *b
24	(Z)-3-Hexen-1-yl butanoate	0	6.1 ± 0.3	nd	nd	nd	6.5 ± 0.3	nd	nd	nd	5.4 ± 0.8	nd	nd
25	Dimethyl tetrasulphide	0	nd	tr	nd	nd	nd	tr	nd	nd	nd	tr	nd
26	β-Cyclocitral	tr	4.9 ± 0.3 a	tr	4.2 ± 0.4 a	tr	tr	tr	tr	tr	tr	tr	tr
27	(Z)-3-Hexen-1-yl 2-methylbutanoate	0	6.4 ± 0.2 a	4.1 ± 0.2 b	2.7 ± 1.1 b	5.2 ± 0.7 b c	6.6 ± 0.4 a	5.5 ± 0.0 c *	5.8 ± 0.1 b	nd	6.3 ± 0.4 a	6.4 ± 0.1 a	2.9 ± 1.5 b
28	(Z)-3-Hexen-1-yl 3-methylbutanoate	0	6.8 ± 0.2 a	3.2 ± 1.3 c	5.6 ± 0.2 b	5.7 ± 0.4 b	6.9 ± 0.2 a	5.6 ± 0.1 b	5.4 ± 0.8 b	5.7 ± 0.4 a	6.1 ± 0.6 a	5.9 ± 0.3 a	5.6 ± 0.0 *a
29	Hexyl 3-methylbutanoate	0	5.4 ± 0.4 a	1.5 ± 0.9 b	nd	nd	nd	nd	nd	nd	tr	nd	nd
30	3-Methyl-2-buten-1-yl hexanoate	0	tr	nd	nd	nd	nd	nd	nd	nd	tr	nd	nd
31	Methyl nerolate	0	nd	6.2 ± 0.1	nd	nd	nd	5.4 ± 0.8	nd	nd	nd	6.3 ± 0.0 *	nd
32	Unidentified	0	4.1 ± 1.1	nd	nd	nd	5.7 ± 0.0 *	nd	nd	nd	nd	nd	nd
33	(Z)-3-hexen-1-yl hexanoate	0	5.7 ± 0.3	nd	nd	nd	6.1 ± 0.4 a	5.3 ± 0.0 *b	nd	nd	5.1 ± 0.7	nd	nd
34	(Z)-β-Elemene	0	5.9 ± 0.1 b	6.2 ± 0.1 a	nd	nd	5.4 ± 0.2 b	5.9 ± 0.2 a	nd	nd	5.6 ± 0.2 b	6.1 ± 0.2 a	nd
35	β-Elemene	0	7.3 ± 0.1 b	7.6 ± 0.1 a	nd	nd	6.7 ± 0.3 b	7.3 ± 0.1 a	nd	nd	7.0 ± 0.2 a	7.4 ± 0.2 a	nd
36	Unidentified (ester)	0	3.7 ± 1.0	nd	nd	nd	tr	nd	nd	nd	tr	nd	nd
37	Octyl 3-methylbutanoate	0	5.3 ± 0.4	nd	nd	nd	4.8 ± 0.5	nd	nd	nd	5.6 ± 0.4	nd	nd
38	(*E*)-β-Farnesene	0	5.4 ± 0.4	nd	nd	nd	5.2 ± 0.7	nd	nd	nd	5.0 ± 0.7	nd	nd
39	β-Ionone	tr	3.8 ± 1.0 a	4.1 ± 0.3 a	4.3 ± 0.5 a	tr	tr	tr	tr	nd	nd	nd	tr
40	α-Acoradiene	0	5.9 ± 0.2 a	6.0 ± 0.1 a	nd	nd	5.4 ± 0.0 *a	5.5 ± 0.1 a	nd	nd	5.6 ± 0.1 a	5.6 ± 0.1 a	nd
41	α-Curcumene	0	5.4 ± 0.5	nd	nd	nd	tr	nd	nd	nd	4.4 ± 0.5	nd	nd
42	β-Selinene	0	4.7 ± 0.5 b	5.6 ± 0.2 a	nd	nd	tr	5.4 ± 0.0 *	nd	nd	4.3 ± 0.4 b	5.6 ± 0.0 *a	nd
43	(Z,*E*)-α-Farnesene	tr	5.4 ± 0.1 a	3.0 ± 0.8 b	5.1 ± 0.3 a	nd	5.5 ± 0.8	tr	2.8 ± 1.4	1.8 ± 1.8 b	5.6 ± 0.5 a	nd	4.9 ± 0.5 a
44	α-Zingiberene	0	6.3 ± 0.2 a	5.9 ± 0.1 b	5.2 ± 0.3 c	nd	5.8 ± 0.3	5.6 ± 0.1	tr	nd	6.0 ± 0.2 a	5.7 ± 0.0 *b	4.2 ± 0.3 c
45	Unidentified	6.0 ± 0.1 a	5.9 ± 0.1 ab	5.6 ± 0.1 c	5.4 ± 0.4 bc	6.4 ± 0.1 a	5.6 ± 0.0 * c	5.9 ± 0.2 b	3.5 ± 1.6 d	6.1 ± 0.1 a	5.6 ± 0.1 b	5.4 ± 0.2 b	4.2 ± 0.4 c
46	(*E*,*E*)-α-Farnesene	6.4 ± 0.2 b	7.2 ± 0.1 a	6.0 ± 0.2 b	7.1 ± 0.1 a	5.4 ± 0.2 c	7.6 ± 0.5 a	tr	6.6 ± 0.2 b	5.9 ± 0.2 b	7.2 ± 0.6 a	tr	6.9 ± 0.2 a
47	(Z)-γ-Bisabolene	0	5.4 ± 0.4	tr	tr	nd	5.6 ± 0.1 a	5.6 ± 0.1 a	tr	nd	4.5 ± 0.6 a	5.0 ± 0.6 a	4.2 ± 0.3 a
48	(*E*)-α-Bisabolene	0	5.1 ± 0.3 a	3.4 ± 0.9 b	nd	nd	4.7 ± 0.4	nd	nd	nd	4.7 ± 0.5	nd	nd
49	(Z)-3-Hexen-1-yl benzoate	0	3.2 ± 1.4	tr	nd	nd	tr	nd	nd	nd	tr	nd	nd
50	TMTT *	0	3.8 ± 1.0	nd	nd	nd	tr	nd	nd	nd	tr	nd	nd
51	Unidentified (hydrocarbon)	3.8 ± 1.0	nd	nd	nd	5.5 ± 0.1	nd	nd	nd	5.5 ± 0.2	nd	nd	nd
52	Unidentified (MS similar to verticilol)	5.6 ± 0.1 a	nd	nd	4.8 ± 0.4 b	6.4 ± 0.1	nd	nd	tr	6.4 ± 0.3 a	nd	nd	5.8 ± 0.1 b

S indicates sulphide; IM—imine; MT—monoterpene; OMT—oxygenated monoterpene; ST—sesquiterpene; HT—homoterpene; IST—isothiocyanate; E—ester; HY—hydrocarbon; RI—retention index; CB—cabbage; CL—cauliflower; CK—curly kale; BR—broccoli; TMTT—(3E,7E)-4,8,12-Trimethyltrideca-1,3,5,7,11-tetraene; RC—reference compound; MS—mass spectrum; values are log-transformed absolute amounts expressed as areas under chromatographic peaks followed by standard error of mean; * indicates that standard error of mean is smaller than 0.05; nd—not detected; tr—trace amount below quantitative limit. Significant differences were calculated between the cultivars within the same plant status, i.e., intact, infested with herbivore and post-damaged, and those values indicated by the same letters are not significantly different (*p* < 0.5, Conover–Iman test with Bonferroni corrections).

## Data Availability

The data used in this study can be provided by the corresponding author upon request.
